# Chemotherapy for advanced non-small cell lung cancer in the elderly population

**DOI:** 10.1590/1516-3180.20161345T1

**Published:** 2016-09-26

**Authors:** Fábio Nasser Santos, Tiago Biachi de Castria, Marcelo Rocha Souza Cruz, Rachel Riera

## Abstract

**BACKGROUND:**

Approximately 50% of patients with newly diagnosed non-small cell lung cancer (NSCLC) are over 70 years of age at diagnosis. Despite this fact, these patients are underrepresented in randomized controlled trials (RCTs). As a consequence, the most appropriate regimens for these patients are controversial, and the role of single-agent or combination therapy is unclear. In this setting, a critical systematic review of RCTs in this group of patients is warranted.

**OBJECTIVES:**

To assess the effectiveness and safety of different cytotoxic chemotherapy regimens for previously untreated elderly patients with advanced (stage IIIB and IV) NSCLC. To also assess the impact of cytotoxic chemotherapy on quality of life.

**METHODS:**

*Search methods:* We searched the following electronic databases: Cochrane Central Register of Controlled Trials (CENTRAL; 2014, Issue 10), MEDLINE (1966 to 31 October 2014), EMBASE (1974 to 31 October 2014), and Latin American Caribbean Health Sciences Literature (LILACS) (1982 to 31 October 2014). In addition, we handsearched the proceedings of major conferences, reference lists from relevant resources, and the ClinicalTrial.gov database.

*Selection criteria:* We included only RCTs that compared non-platinum single-agent therapy versus non-platinum combination therapy, or non-platinum therapy versus platinum combination therapy in patients over 70 years of age with advanced NSCLC. We allowed inclusion of RCTs specifically designed for the elderly population and those designed for elderly subgroup analyses.

*Data collection and analysis:* Two review authors independently assessed search results, and a third review author resolved disagreements. We analyzed the following endpoints: overall survival (OS), one-year survival rate (1yOS), progression-free survival (PFS), objective response rate (ORR), major adverse events, and quality of life (QoL).

**MAIN RESULTS:**

We included 51 trials in the review: non-platinum single-agent therapy versus non-platinum combination therapy (seven trials) and non-platinum combination therapy versus platinum combination therapy (44 trials).

Non-platinum single-agent versus non-platinum combination therapy

Low-quality evidence suggests that these treatments have similar effects on overall survival (hazard ratio (HR) 0.92, 95% confidence interval (CI) 0.72 to 1.17; participants = 1062; five RCTs), 1yOS (risk ratio (RR) 0.88, 95% CI 0.73 to 1.07; participants = 992; four RCTs), and PFS (HR 0.94, 95% CI 0.83 to 1.07; participants = 942; four RCTs). Non-platinum combination therapy may better improve ORR compared with non-platinum single-agent therapy (RR 1.79, 95% CI 1.41 to 2.26; participants = 1014; five RCTs; low-quality evidence).

Differences in effects on major adverse events between treatment groups were as follows: anemia: RR 1.10, 95% 0.53 to 2.31; participants = 983; four RCTs; very low-quality evidence; neutropenia: RR 1.26, 95% CI 0.96 to 1.65; participants = 983; four RCTs; low-quality evidence; and thrombocytopenia: RR 1.45, 95% CI 0.73 to 2.89; participants = 914; three RCTs; very low-quality evidence. Only two RCTs assessed quality of life; however, we were unable to perform a meta-analysis because of the paucity of available data.

Non-platinum therapy versus platinum combination therapy

Platinum combination therapy probably improves OS (HR 0.76, 95% CI 0.69 to 0.85; participants = 1705; 13 RCTs; moderate-quality evidence), 1yOS (RR 0.89, 95% CI 0.82 to 0.96; participants = 813; 13 RCTs; moderate-quality evidence), and ORR (RR 1.57, 95% CI 1.32 to 1.85; participants = 1432; 11 RCTs; moderate-quality evidence) compared with non-platinum therapies. Platinum combination therapy may also improve PFS, although our confidence in this finding is limited because the quality of evidence was low (HR 0.76, 95% CI 0.61 to 0.93; participants = 1273; nine RCTs).

Effects on major adverse events between treatment groups were as follows: anemia: RR 2.53, 95% CI 1.70 to 3.76; participants = 1437; 11 RCTs; low-quality evidence; thrombocytopenia: RR 3.59, 95% CI 2.22 to 5.82; participants = 1260; nine RCTs; low-quality evidence; fatigue: RR 1.56, 95% CI 1.02 to 2.38; participants = 1150; seven RCTs; emesis: RR 3.64, 95% CI 1.82 to 7.29; participants = 1193; eight RCTs; and peripheral neuropathy: RR 7.02, 95% CI 2.42 to 20.41; participants = 776; five RCTs; low-quality evidence. Only five RCTs assessed QoL; however, we were unable to perform a meta-analysis because of the paucity of available data.

**AUTHORS' CONCLUSIONS:**

In people over the age of 70 with advanced NSCLC who do not have significant co-morbidities, increased survival with platinum combination therapy needs to be balanced against higher risk of major adverse events when compared with non-platinum therapy. For people who are not suitable candidates for platinum treatment, we have found low-quality evidence suggesting that non-platinum combination and single-agent therapy regimens have similar effects on survival. We are uncertain as to the comparability of their adverse event profiles. Additional evidence on quality of life gathered from additional studies is needed to help inform decision making

The abstract, the full text of this review (English) and a plain language summary (for patients and consumers, in English, Croatian, German and Russian) are available from: http://onlinelibrary.wiley.com/doi/10.1002/14651858.CD010463.pub2/full
